# Antibody Prevalence of West Nile Virus in Birds, Illinois, 2002

**DOI:** 10.3201/eid1006.030644

**Published:** 2004-06

**Authors:** Adam M. Ringia, Bradley J. Blitvich, Hyun-Young Koo, Marshall Van de Wyngaerde, Jeff D. Brawn, Robert J. Novak

**Affiliations:** * Illinois Natural History Survey, Champaign, Illinois, USA;; † Colorado State University, Fort Collins, Colorado, USA;; ‡ University of Illinois, Champaign, Illinois, USA

**Keywords:** ELISA, epidemiology, flavivirus, Illinois, serology, West Nile virus

## Abstract

Antibodies to West Nile virus were detected in 94 of 1,784 Illinois birds during 2002. Captive and urban birds had higher seropositivity than did birds from natural areas, and northern and central Illinois birds’ seropositivity was greater than that from birds from the southern sites. Adult and hatch-year exposure rates did not differ significantly.

West Nile virus (WNV; family *Flaviviridae*, genus *Flavivirus*) was first identified in the Western Hemisphere in 1999 (*1*) and had been detected in 27 states of the United States by the end of 2001 (*2*). Despite abundant evidence of avian, mosquito, and mammalian transmission (*2*), few reports are available on the exposure of live birds to WNV outside of New York and New Jersey.

WNV activity was first detected in Illinois in September 2001 (*3*). During 2001, its distribution was limited to seven counties, primarily in northeastern Illinois (*3*). In 2002, however, Illinois had the greatest number of human WNV cases in the country (884 cases, 66 deaths) as well as reports of WNV infections in mammals, mosquitoes, and dead birds from all but two counties (*3*). Prior to and concurrent with this outbreak, we collected blood samples from both wild and domestic birds to compare exposure rates among species, geographic regions, and urban and natural habitats.

## The Study

**Figure 1:**
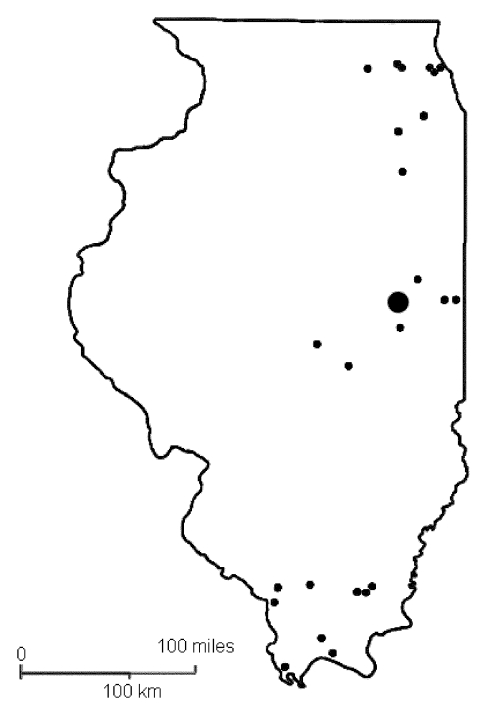
Locations of the study sites in the avian serologic survey for West Nile virus infection, Illinois, 2002.

Wild birds were collected from 43 study sites in Illinois (Figure 1) from February through December 2002 by using standard methods (*4*). Sites were classified as urban (agricultural, industrial, and residential), natural (forested areas, woodlots, and wetlands), or captive (locations where birds were confined). All captured birds were identified to species and, when possible, by sex and as adult or hatch-year (*5**,**6*). Before release, all birds were marked with fingernail polish on the tarsus and retrices to prevent repeated sampling of the same bird within a short period. Captive birds were collected from six study sites (one northern, three central, and two southern locations).

Serum samples were tested for antibodies to WNV by epitope-blocking enzyme-linked immunosorbent assay (ELISA), according to the protocols of Blitvich et al. (*7*). ELISAs were performed with two monoclonal antibodies (MAbs), 3.1112G and 2B2. Recent studies have shown that assays performed with these MAbs detect antibodies to WNV in taxonomically diverse North American avian species (*7*). Furthermore, assays performed with MAb 3.1112G discriminate between WNV and St. Louis encephalitis virus infections in birds. The ability of the Illinois bird sera to block the binding of the MAbs to WNV antigen was compared to the blocking ability of normal chicken serum (Vector Laboratories, Burlingame, CA). The percentage inhibition value was calculated as previously described (*7*). Any serum sample that blocked the binding of both MAbs by >30% was considered positive for antibodies to WNV. We required a positive result from both MAbs because of our lack of access to plaque reduction neutralization testing.

Serum samples were collected from 1,784 birds, representing 10 orders and 81 species. In total, 94 birds, representing 5 orders and 19 species, were positive for antibodies to WNV (Table 1). The overall exposure rate for the year was 5.3%. The species with the highest seropositivity (>10% and >1 positive sample) were Rock Doves, Great Horned Owls, Chukar, Northern Cardinals, House Sparrows, and Brown Thrashers.

**Table 1:** Birds, listed alphabetically by order, tested for WNV antibody in Illinois in 2002, including number of birds positive and number tested^a^

Order	Common name	No. tested	No. WNV-positive (%)	95% CI
Anseriformes	Canada Goose	253	3 (1.2)	0.3 to 3.4
	Wood Duck	120	3 (2.5)	0.5 to 7.1
	3 additional species	35	0	
Columbiformes	Mourning Dove	11	1 (9.1)	0.2 to 41.3
	Rock Dove^b^	20	11 (55.0)	31.5 to 76.9
Galliformes	Chukar^b^	22	6 (27.3)	10.7 to 50.2
	Domestic Chicken^b^	63	5 (7.9)	2.6 to 17.6
	2 additional species	16	0	
Passeriformes	Cedar Waxwing	5	1 (20.0)	0.5 to 71.6
	Blue Grosbeak	2	1 (50.0)	1.2 to 98.7
	Indigo Bunting	28	1 (3.6)	0.1 to 18.4
	Northern Cardinal	129	16 (12.4)	7.3 to 9.4
	American Crow	157	5 (3.2)	1.0 to 7.3
	Red-winged Blackbird	39	3 (7.7)	1.6 to 20.9
	Brown Thrasher	19	2 (10.5)	1.3 to 33.1
	Gray Catbird	72	6 (8.3)	3.1 to 17.3
	Ovenbird	32	1 (3.1)	0.1 to 16.2
	House Sparrow	185	21 (11.4)	7.1 to 16.8
	American Robin	79	3 (3.8)	0.8 to 10.7
	Swainson’s Thrush	32	1 (3.1)	0.1 to16.2
	45 additional species	422	0 (0)	
Strigiformes	Great Horned Owl^b^	9	4 (44.4)	13.7 to 78.8
	2 additional species	3	0	
Other (5 orders)	10 species	31	0	
Total (10 orders)	81 species	1784	94 (5.3)	4.2 to 6.4

^a^WNV, West Nile virus; CI, confidence interval. ^b^Indicates captive specimens.

We determined the relative importance of region (north/central/south), habitat (urban/natural), and month of capture to antibody prevalence by using stepwise logistic regression (8; Table 2). This model explains 14% of the variation in antibody prevalence.

**Table 2:** Logistic regression analysis of Illinois avian WNV antibody prevalence, 2002^a^

Factor	DF	Wald χ^2^	p value
Region	2	17.65	<0.0001
Month	10	44.80	<0.0001
Habitat (urban/natural)	1	1.29	0.26
Full model	13	78.21	<0.0001
r^2^ = 0.14			

^a^WNV, West Nile virus; DF, degrees of freedom. ^b^Dependent variable, presence or absence of WNV antibody.

Our first seropositive bird was captured on April 26, 2002, in central Illinois. In some birds, immunoglobulin (Ig) M and IgG antibodies are not detectable for 4 to 5 days, and then build to a peak at 7 to 8 days or 3–4 weeks, respectively, before antibody levels start to decline (*9*). This pattern suggests that transmission in Illinois occurred at least as early as mid-April. However, the specific immune response to WNV is unknown in most bird species.

**Figure 2:**
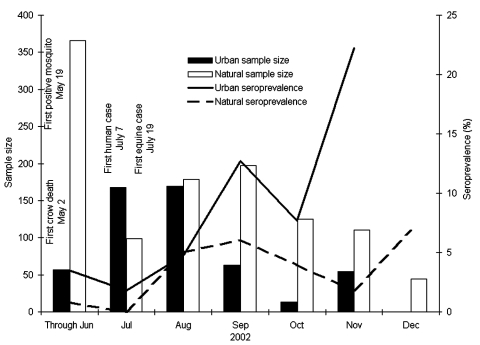
Monthly percentage of West Nile virus antibody–positive birds in Illinois during 2002, with corresponding sample size. First human, mosquito, and equine cases for Illinois are reported for comparison. Bars show the monthly sample size and lines indicate the monthly seroprevalence.

Avian movement can have a major impact on the measured temporal exposure rates of birds. Although antibody-positive rates increased steadily beginning in August, prevalence decreased during October (Figure 2). This decrease corresponds with the time when many birds are migrating into and through Illinois (*10*). If these birds are moving from areas of lower transmission, the proportion of antibody-positive birds may have been reduced. Additionally, WNV antibody prevalence was highest in both urban and natural settings in the final collections of 2002, after many of the migrants had moved on. We speculate that the increased rate of seropositive birds in the winter was the result of the changing geographic distribution of birds in the winter rather than continuing winter transmission, despite the rare winter detection of virus in raptors (*11*).

We compared antibody prevalence by habitat type, geographic region, and age separately using chi-square analysis (Table 3). The prevalence of antibodies to WNV was significantly higher in birds from northern and central Illinois than in those from southern Illinois. The north-south gradient in temperature, vegetation, topography, human population density, and land use could all influence regional transmission dynamics. Also, both birds and mosquito species vary across the state (*6**,**12*). Any of these factors may contribute to the differences that we report.

**Table 3:** Differences in West Nile virus seropositivity in birds by age, region, and habitat using chi-square analysis, Illinois, 2002

Comparison		Samples, n (% total); N = 1,784	Antibody + (%)^a^
Habitat	Urban	524 (29.4%)	34 (6.49%)^A^
	Natural	1,121 (62.8)	34 (3.03%)^B^
	Captive	139 (8.2%)	26 (18.71%)^C^
		χ^2^ = 63.06	p < 0.0001
Region	Northern	412 (23.1%)	20 (4.85%)^D^
	Central	796 (44.6%)	62 (7.79%)^D^
	Southern	576 (32.3%)	12 (2.08%)^E^
		χ^2^ = 21.98	p < 0.0001
Age^b^	Adult	455 (25.5%)	10 (2.2%)^F^
	Hatch-year	508 (28.5%)	21 (4.1%)^F^
		χ^2^ = 2.81	p < 0.096

^a^Like (capital letter) superscripts indicate no difference in pairwise comparisons. ^b^821 of the 1,784 birds had no age recorded at time of collection. The remaining 963 were used for the age analysis.

Overall, birds from urban areas were more commonly seropositive than birds from natural sites (Table 3). A likely explanation for this result is that *Culex pipiens*, the primary vector of WNV in Illinois, is closely associated with human environments (*12*). Also, captive species showed higher rates of exposure to WNV than birds in either urban or natural habitats (Table 3). In fact, among the species most frequently infected were Rock Doves, Chukar, and Great Horned Owls, all of which are captive species. Captive birds are housed in unnatural conditions that may facilitate their exposure to WNV infection by increased bird density, increased bird-to-bird transmission from contact with sick or injured birds, or their inability to escape from mosquitoes (*13*). Many of the serum specimens from Great Horned Owls, for example, were collected from sick birds that had been turned in to wildlife rehabilitators. Therefore, we suspect that the seroprevalence values of WNV in captive birds may not be representative of the infected proportion of those species found in the wild. House Sparrows, Brown Thrashers, and Northern Cardinals, the free-ranging species with the highest antibody prevalence, are all locally abundant birds, which increases the probability of their contact with infected mosquitoes. Although many of the species with high exposure rates are common birds in urban areas (House Sparrows, Cardinals), others (Brown Thrashers, Gray Catbirds) are more frequently associated with natural habitats, which suggests that WNV transmission occurred in both habitat types. Our serologic results and the reservoir competence studies of Komar et al. (*14*) indicate that members of the families *Cardinalidae* and *Mimidae* are good candidates for reservoir competence testing. We speculate that the variation in seroprevalence is the result of a combination of factors, including defensive behaviors, host preference of mosquitoes, habitat association, and roosting behaviors.

American Crows were rarely seropositive, despite the collection of crows exhibiting WNV symptoms. Several of these crows were subsequently found to be WNV positive on necropsy (RJ Novak, unpub. data), supporting the findings of Komar et al. (*14*) that American Crows and Blue Jays frequently die 4–6 days postinfection, which is before antibodies are detectable in some species (*9*). This finding suggests that antibody prevalence may not be correlated with the impact of WNV on population numbers in some species.

We found no significant difference in the proportion of adult and hatch-year birds with antibodies to WNV (Table 3), which supports the finding of Komar et al. (*15*) that that pattern is normal for virus activity in a new location. We did not detect antibodies to WNV in any birds captured before late April, which suggests that limited or no WNV transmission occurred before or during the winter of 2001 in Illinois.

Although WNV was first reported in Illinois in 2001, statewide WNV activity was not detected until 2002. The mechanisms for both the short- and long-distance dispersal of WNV are not fully understood. Migrating birds are suspected of playing a major role in the long-distance dispersal of WNV into new areas (*16*). In our collections, we found only one seropositive bird that does not nest or winter in Illinois, a Swainson’s Thrush, captured on August 28, 2002.

## Conclusions

WNV infections were detected in numerous mosquito pools, dead birds, equines, and >800 humans in Illinois in 2002, with virus activity reported in almost every county (*3*). However, the overall avian seroprevalence (5.3%) of WNV in the present study was low. Similarly, low WNV infection rates were reported in birds during the New York epizootics of 2000 and 2001 (6.9% and 7.0, respectively 17,18; ). However, several species exhibited exposure rates >10%.

Our data demonstrate the great diversity of avian species that are susceptible to WNV infection, a finding consistent with earlier studies (*19*). Although transmission rates and corresponding variation in seroprevalence may be related to defensive behaviors, grouping, or habitat associations, our results show that captive birds and those in urban areas are more likely to be infected than those in natural areas. Dead bird surveillance is typically limited to corvids (Blue Jays and Crows). However, live bird serosurveys clearly demonstrate the broad range of avian species exposed to WNV. The impact of WNV on the illness and death of most of these species remains unknown. Therefore, continued research is required to understand the complex transmission patterns and epidemiologic impact of WNV.
